# Is transverse screw fixation really necessary in PAO?—A comparative *in vivo* study

**DOI:** 10.1093/jhps/hnab034

**Published:** 2021-05-03

**Authors:** Vincent J Leopold, Juana Conrad, Christian Hipfl, Maximilian Müllner, Thilo Khakzad, Carsten Perka, Sebastian Hardt

**Affiliations:** Department of Orthopaedic Surgery and Traumatology, Charité Berlin, University Hospital, Chariteplatz 1, Berlin 10117, Germany

## Abstract

The optimal fixation technique in periacetabular osteotomy (PAO) remains controversial. This study aims to assess the *in vivo* stability of fixation in PAO with and without the use of a transverse screw. We performed a retrospective study to analyse consecutive patients who underwent PAO between January 2015 and June 2017. Eighty four patients (93 hips) of which 79% were female were included. In 54 cases, no transverse screw was used (group 1) compared with 39 with transverse screw (group 2). Mean age was 26.5 (15–44) in group 1 and 28.4 (16–45) in group 2. Radiological parameters relevant for DDH including lateral center edge angle of Wiberg (LCEA), Tönnis angle (TA) and femoral head extrusion index (FHEI) were measured preoperatively, post-operatively and at 3-months follow-up. All patients were mobilized with the same mobilization regimen. Post-operative LCEA, TA and FHEI were improved significantly in both groups for all parameters (*P* ≤ 0.0001). Mean initial correction for LCEA (*P* = 0.753), TA (*P* = 0.083) and FHEI (*P* = 0.616) showed no significant difference between the groups. Final correction at follow-up of the respective parameters was also not significantly different between both groups for LCEA (*P* = 0.447), TA (*P* = 0.100) and FHEI (*P* = 0.270). There was no significant difference between initial and final correction for the respective parameters. Accordingly, only minimal loss of correction was measured, showing no difference between the two groups for LCEA (*P* = 0.227), TA (*P* = 0.153) and FHEI (*P* = 0.324). Transverse screw fixation is not associated with increased fragment stability in PAO. This can be taken into account by surgeons when deciding on the fixation technique of the acetabular fragment in PAO.

## INTRODUCTION

Developmental dysplasia of the hip (DDH) is a complex pathology and a leading cause of secondary osteoarthritis of the hip [1–4]. If diagnosed in time, joint-preserving surgical therapy with periacetabular osteotomy (PAO) is a good treatment option in young adults with symptomatic DDH showing good to excellent outcomes both clinically and radiologically [5–9]. The surgical technique has been well described before [5, 10]. The goal of PAO is the redirection of the misaligned acetabulum to a position that provides improved coverage of the femoral head with hyaline cartilage [5, 10]. In PAO, the acetabulum is completely detached from the pelvis through 5 osteotomies and can thus be reoriented threedimensionaly. After adequate reorientation, the acetabular fragment is fixed in place to provide better coverage of the femoral head. In the original description of the technique this definitive fixation is achieved through two screws introduced from the iliac crest into the acetabular fragment and one additional horizontal screw. This horizontal screw is inserted in the anteroposterior (AP) direction through the fragment into the ilium aiming at the sacroiliac joint [10]. Since its first description, the technique has been established worldwide and is currently performed at many centers. As the technique became more widespread, different forms of fixation of the reoriented acetabular fragment were described. Besides the original technique mentioned above, fixations without the use of a horizontal screw have been reported [5, 10–18]. Various studies have compared different fixation techniques with and without horizontal screws [12–15]. However, all of these studies were in vitro studies. *In vivo* studies comparing the stability of fixation techniques with and without transverse screws are lacking. This study aims to assess the fixation stability of screw fixation—with and without use of a horizontal screw—in patients undergoing PAO for DDH.

## MATERIALS AND METHODS

### Demographics

**Fig. 1.**
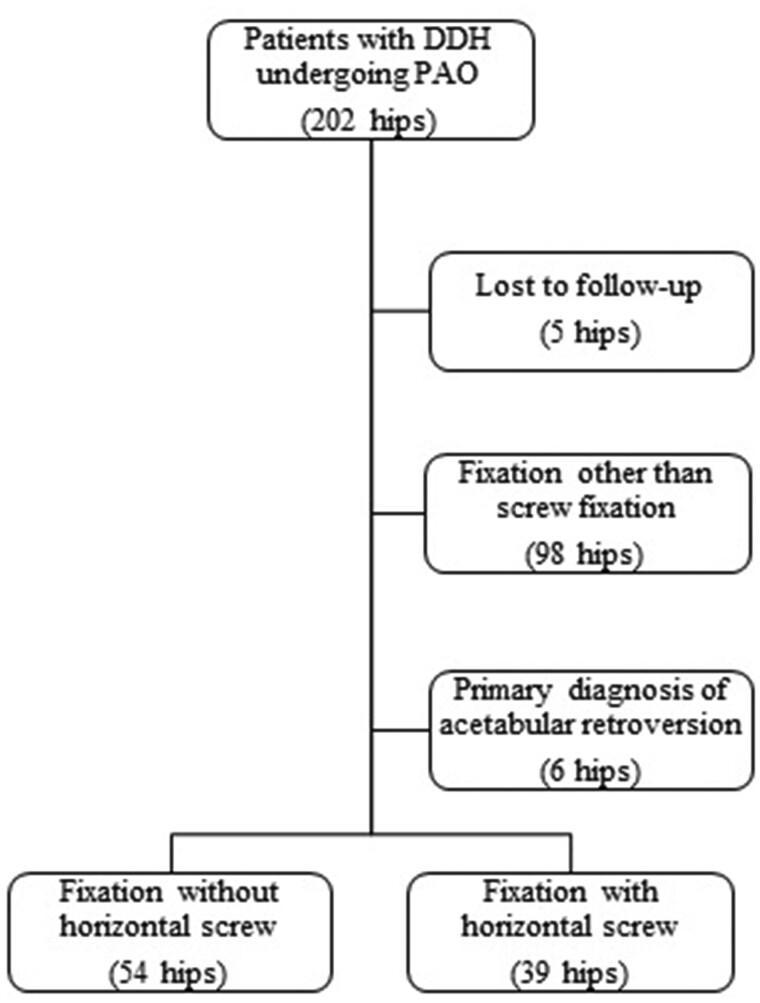
Patient selection.

We performed a retrospective study of 183 patients (202 hips) with a primary diagnosis of DDH undergoing PAO at our institution between January 2015 and June 2017. Approval from the local ethics committee was obtained beforehand. Inclusion criteria were patients with adequate radiological imaging pre- and post-operatively and at least 3 month follow-up. Exclusion criteria were primary diagnosis other than DDH, such as acetabular retroversion, prior operation on the ipsilateral hip joint and fixations other than screw fixation. In 5 cases, no radiological imaging at 3 month follow up was available leaving a final study cohort of 84 patients (93 hips) of whom 79% were female. An overview of patient selection is shown in Fig. 1.

All hips featured at least one radiological anomaly, including a lateral center edge angle of Wiberg (LCEA) of <25° [12], Tönnis angle (TA) of >10° [12], anterior center edge angle (ACE) according to Lequesne and de Seze of <25° [13] and a femoral head extrusion index (FHEI) according to Heyman and Herndeon of 26% or more [14]. Femoral head congruence was determined preoperatively by 30° abduction functional radiographs and was good in all hips. The demographic data collected included age, gender, and body mass index (BMI). The demographic data are shown in Table I.

**Table I.** Demographics

	No horizontal screw (54 hips)	Horizontal screw (39 hips)	*P*-values
Age	26.50 (SD 8.80 range 15–44)	28.41 (SD 8.19; range 16–45)	0.291*
BMI	23.81 (SD 4.68; range 16.3–35.9)	25.43 (SD 4.39; range 18.3–35.8)	0.134*
Male/female	9/45	11/28	0.208**
Follow up (days)	82.04 (SD 11.03 range 67–101)	81.53 (SD 10.69 range 70–103)	0.883*

Values are presented as mean with standard deviation and range (* unpaired *t* test;

** Fishers exact test).

### Surgical technique

**Fig. 2.**
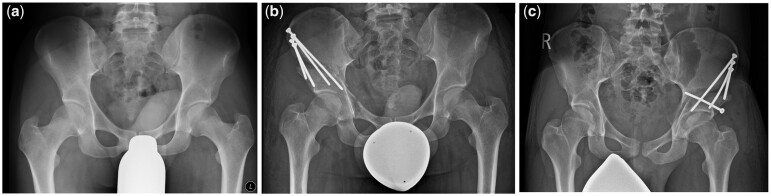
(**a**) Preoperative radiograph. (**b**) Post-operative radiograph after PAO without transverse screw fixation. (**c**) Post-operative radiograph this time with use of a transverse screw.

All PAOs were performed by two experienced surgeons at our clinic. Fixation with (*n* = 39) or without (*n* = 54) the use of horizontal screw fixation was performed on the surgeon’s discretion (Fig. 2). The surgical technique of PAO has been described before [5, 10]. All operations were performed through an anterior approach under fluoroscopic guidance. The acetabulum was liberated and reoriented through 5 osteotomies. After reorientation, the acetabular fragment was temporarily fixed with Kirschner wires. After subsequent fluoroscopic control, a further correction was performed if necessary. The final fixation was then performed with 4.5 mm titanium screws. Either 3–4 screws were introduced through the anterior superior iliac spine (ASIS) into the acetabular fragment or 2–3 screws were introduced through the ASIS adding a single horizontal screw through the acetabular fragment into the ilium aiming at the sacroiliac joint. In both fixation groups the total number of screws was based on the patient’s anatomy and surgeon’s discretion. All screws were 4.5 mm, fully threaded, and cortical. The target of intraoperative reorientation was defined as normalization of LCEA >30°, AI <10° and FHEI between 10% and 25%. The post-operative mobilization regimen was the same in both groups. All patients were mobilized in tip-touch partial weight-bearing for a period of 6 weeks post-operatively. After week 6 weight-bearing was increased to half of the patient’s body weight from the seventh to the 10th post-operative week. After week 10 weight-bearing was then gradually increased to full weight-bearing until 3 months post-operatively. No restriction was imposed on the range of motion of the operated hip joint.

### Radiological assessment

All patients received standardized standing AP pelvis radiographs. Radiological parameters relevant for DDH were measured preoperatively, post-operatively before discharge and at 3-month follow-up. This time was chosen for follow-up because the patients had reached full weight bearing of the operated extremity at this point in time according to our mobilization regime. A loss of reposition would therefore be most likely to be expected during this period and thereafter considered less likely. Radiological parameters measured were LCEA, TA as well as FHEI. All measurements were performed by two residents (VL, JC), both trained by the same senior orthopedic surgeon (SH). A loss of correction was defined as the difference between initial correction at immediate post-operative time and at 3 months follow up. Significant loss of correction was defined as a loss of acetabular fixation that required an additional surgical procedure or a delta LCEA of >5° as measured in the radiological evaluation. The presence or absence of non-unions was determined by evaluation of bony consolidation along the osteotomies. Complete or incomplete implant removal after implant extraction surgery was evaluated on the basis of the radiographs as well as the surgical reports.

### Statistical analysis

Intra- and inter-rater reliability was assessed using an intra-class correlation coefficient (ICC) model. Kappa value was used to confirm intra- and inter-rater reliability. Frequency rates, means, and range were utilized to describe baseline patient characteristics. *T* test was used to determine significant differences between continuous data and Fishers exact test for categorical data. A *P* value of <0.05 was considered statistically significant. For documentation of the collected data, Microsoft Excel version 16.16.2 was used. The collected data were analysed using IBM SPSS 27.

## RESULTS

**Fig. 3.**
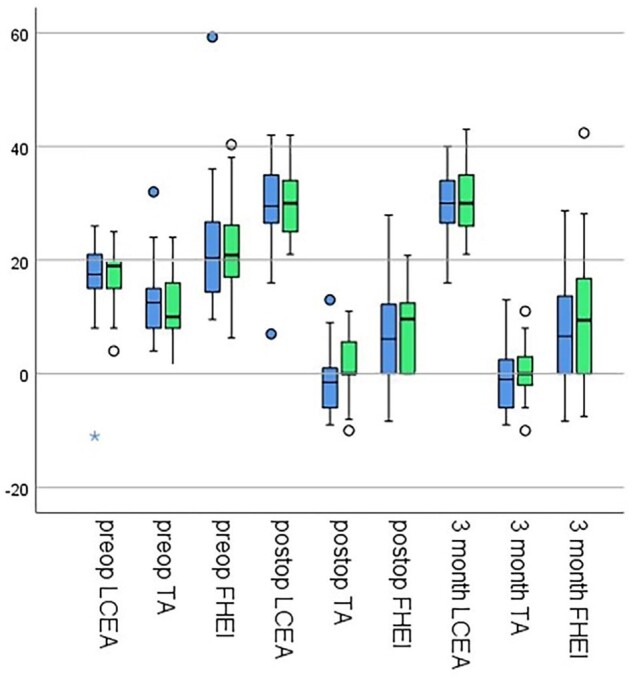
Comparison of radiological parameters for both groups preoperatively, post-operatively and at 3-month follow up. Blue: No transverse screw. Green: Transverse screw used.

The inter- and intra-observer reliabilities for the measurements of radiologic parameters were excellent for all parameters, ranging from 0.97 to 0.98. There was no significant difference observed regarding preoperative radiologic parameters between the two groups for LCEA (*P* = 0.649), TA (*P* = 0.541), or FHEI (*P* = 0.454). The post-operative LCEA, TA and FHEI were improved significantly in both groups for all parameters (*P* ≤ 0.0001). The mean initial correction for LCEA (*P* = 0.753), TA (*P* = 0.083), and FHEI (*P* = 0.616) showed no significant difference between the two groups. The final correction of the respective parameters was also not significantly different between both groups for LCEA (*P* = 0.447), TA (*P* = 0.100), and FHEI (*P* = 0.270). Within the respective groups there was no significant difference between initial and final correction for the LCEA (0.901 versus 0.106), TA (0.562 versus 0.061), and FHEI (0.966 versus 0.286) (Fig. 3). Accordingly, no significant loss of correction was measured, showing no difference between the two groups for LCEA (*P* = 0227), TA (*P* = 0.153), and FHEI (*P* = 0.324). Overall, there were no cases in either fixation group with significant loss of correction as measured by radiographs or that led to reoperation. No cases of non-union were found in either group. Overall in 34/93 (36.6%) cases, patients complained about soft tissue irritation caused by the osteosynthesis material and were therefore admitted to implant extraction surgery. In the group without use of a transverse screw, implant removal was performed in 23/54 cases (42.6%) compared with 11/39 cases (28.8%) in the group with a transverse screw, showing no significant difference between the two groups (*P* = 0.193). However in the first group there was no case of partial implant removal (0%) compared with 7 cases (63.6%) in the second group (*P* = 0.001). In all of these cases, the more complex preparation with additional or longer incision necessary for removal of the transverse screw was discussed with the patients prior to surgery and it was mutually decided to leave the transverse screw in place. An overview of the measured radiological parameters is shown in Table II.

**Table II.** Comparison *of correction of LCEA, TA* *and FHEI between the two groups*

	No horizontal screw (54 hips)	Horizontal screw (39 hips)	*P*-values
LCEA (°)			
Pre-operative	17.91 (SD 6.49; range −11 to 26)	17.32 (SD 5.55; range −1 to 25)	0.649*
Post-operative	31.13 (SD 6.45; range 7–42)	30.51 (SD 5.78; range 18–42)	0.636*
Initial correction	13.22 (SD 5.37; range 3–26)	13.64 (SD 7.42; range 5–39)	0.753*
Final correction	12.97 (SD 5.11; range 3–27)	14.36 (SD 8.67; range 5–43)	0.447*
Loss of correction	0.06 (SD 2.82; range −5 to 9)	1.00 (SD 3.01; −6 to 9)	0.227*
Tönnis angle (°)			
Pre-operative	11.93 (SD 6.20; range −2 to 32)	11.11 (SD 6.47; range −1 to 24)	0.541*
Post-operative	−1.25 (SD 5.90; range −14 to 13)	0.233 (SD 6.50; range −13 to 11)	0.252*
Initial correction	−13.18 (SD 6.90; range −31 to 2)	−10.58 (SD 7.24; range −24 to 7)	0.083*
Final correction	–13.90 (SD 6.23 range −31 to (−4))	–10.91 (SD 7.63; range −24 to 7)	0.100*
Loss of correction	−0.25 (SD 2.40; range −7 to 5)	−1.17 (SD 2.43 range −6 to 7)	0.153*
FHEI (%)			
Pre-operative	21.03 (SD 9.06; range 9.55–59.24)	22.35 (SD 7.28; range 6.31–40.32)	0.454*
Post-operative	7.46 (SD 7.74; range −8.38 to 27.9)	9.54 (SD 7.07 range −5.81 to 20.82)	0.188*
Initial correction	–13.57 (SD 6.62; range −31.34 to (−0.13)	−12.81 (SD 7.86; range −40.32 to 1.81)	0.616*
Final correction	−14.18 (SD 7.08; range −30.59 to (−3.65))	−11.64 (SD 10.22; range −40.32 to (−0.52)	0.270*
Loss of correction	0.035 (SD 4.74 range −13.95 to 11.94)	1.66 (SD 7.48; range −18.90 to 21.55)	0.324*

Values are presented as mean, standard deviation and range (* unpaired *t* test).

## DISCUSSION

PAO is an established procedure for joint-preserving surgical therapy of DDH in young adults. Different fixation techniques of the acetabular fragment are established. The original technique using a transverse screw is being used worldwide. Several studies exist, investigating the biomechanical in vitro stability of different techniques with and without transverse screw. To our knowledge, no study has previously compared the *in vivo* stability of fragment fixation with and without use of a transverse screw in patients undergoing PAO.

The most important finding of the presented study was that there was no difference between the two investigated techniques in terms of fixation stability and loss of correction. There was also no difference observed in terms of osteotomy healing. Implant extraction surgery was performed at comparable frequencies, with only partial implant removal being significantly more frequent in the transverse screw group.

In the investigated cohort, no difference was observed between the groups regarding the preoperatively measured radiological parameters. There was also no significant difference between the two groups with respect to the extent of the correction. Both groups were corrected to a comparable degree and showed almost identical correction values for initial correction immediately after surgery and final correction at the time of follow-up (see Table II). This resulted in almost no loss of correction, which did not differ significantly between the two groups. There were also no cases of non-union observed in either group in this study.

Several studies have previously assessed the biomechanical stability of different fixation constructs under in vitro conditions. A study by Babis et al. compared the stability of the fixation forms of 3 screws inserted through the ASIS with that of 2 screws inserted through the ASIS into the fragment and an additional transverse screw in an in vitro cadaver pelvis model simulating the push-off phase of the gait cycle. The authors found a higher stiffness and higher absolute values for catastrophic failure loads in the transverse screw group (930.8 ± 306.5 N) compared with the group without one (741.5 ± 286.0 N). These differences showed a tendency but no significance. The catastrophic failure loads shown here were at 1.27 times the body weight in the group without the use of a transverse screw compared with 1.66 times the body weight in the group with transverse screw. These values are relatively low considering that during gait at normal pace the peak loads on the hip joint are ∼2.8 times the body weight and ∼5.5 times during periods of instability in one-leg-stand [19]. These values apply to the proximal femur in the hip joint region. However, another study by Pedersen et al. was able to show that the peak values of joint force acting in the periacetabular region are similar to those previously described for the proximal femur [20]. It can therefore be stated that both fixation methods are not suitable for immediate post-operative full weight bearing. Another study by Widmer et al. used a comparable in vitro setup simulating the push-off phase of the normal gait cycle also comparing fixation constructs with and without use of a transverse screw. The authors found favorable results for the transverse screw construct showing higher failure loads here. With the use of sawbones pelves in this study, the preconditions for conclusions were limited [12].

Another study by Bergmann et al. shows that already during normal gait forces between 1925 and 2880 N are acting on the hip joint. Comparing these values with the aforementioned failure loads reported by Babis et al., supports the presumption that full weight-bearing in the early post-operative phase cannot be recommended [13, 21].

Instead the previously described mobilization regimen with post-operative tip-touch partial weight bearing of the operated leg and gradual increase of weight-bearing thereafter was applied. Generally after PAO, patients are instructed to mobilize in tip-touch partial weight-bearing for a period of 6–10 weeks to avoid loss of correction or failure of fixation. Similar loading regimes are commonly applied [22, 23] and were also described by the inventors of the technique [10].

In our cohort there was no significant loss of correction in either group under the described mobilization regimen in the immediate post-operative phase until follow-up. Follow-up was set at 3 months post-operatively, with patients having reached full weight bearing of the operated leg at that time. Without a significant loss of correction to be found in the analysed radiographs at this stage, the assumption can be made that loss of correction thereafter is unlikely.

When taking a closer look at the literature regarding osseous healing after PAO it can be found that more rigid fixation constructs are associated with significantly higher rates of non-unions in [24–26]. In our cohort no case of non-union was observed in either group. However, according to the aforementioned studies the probability for non-unions without the use of a transverse screw seems to be lower theoretically as it was shown that a transverse screw adds rigidity to the fixation [13].

Another factor in the decision for or against the use of a transverse screw can be pending implant removal surgery. Implant removal is frequently performed following PAO due to soft tissue irritation and the patients request [24, 27, 28]. This was also observed in our cohort as in 36.6% of the cases implant removal was performed. Implant removal is more complex if a transverse screw was used and the material is to be removed completely, since a longer or second incision with additional, soft tissue preparation is necessary to address the transverse screw. Furthermore, retained metal implants may pose a problem after PAO, considering possible need of MRI in the future. Even though artifact reduction techniques have improved, metal screws will produce a lot of artifact in this process [29, 30]. In addition, remaining implants may cause a challenge in eventual conversion to THA, which still becomes necessary in a relevant proportion of patients after PAO [7].

There are several limitations to the present study. First, its retrospective study design and relatively small sample size leads to limited statistical power. Second, the operations in the study were performed by two different surgeons, which may lead to potential statistical bias. Third, it is not possible to deduce how the examined fixations behave under higher loads in the early post-operative phase. However, the post-operative weight-bearing regimen was not the subject of this study. Fourthly, the follow-up at 3 months was by definition too early to assess the presence of non-unions. Nevertheless, with radiologically proven bony consolidation of the osteotomies and painless mobilization under full weight-bearing, we assumed the absence of non-unions at that time. Despite these limitations, this study is the first series comparing the efficacy of PAO with and without the use of a transverse screw.

In conclusion, this study demonstrated that acetabular fragment fixation without use of a transverse screw is a safe and viable option in PAO with comparable stability, provided that a standardized mobilization regimen with partial weight-bearing is applied in the immediate post-operative phase. Under these circumstances, transverse screw fixation does not seem to be necessary. This can be taken into account by surgeons when deciding on the fixation technique of the acetabular fragment in PAO.
